# Search strategies along the academic lifecycle

**DOI:** 10.1007/s11192-012-0789-3

**Published:** 2012-06-16

**Authors:** Edwin Horlings, Thomas Gurney

**Affiliations:** Science System Assessment Department, Rathenau Institute, The Hague, The Netherlands

**Keywords:** Mapping science, Academic careers, Lifecycle, Agenda setting, Problem choice, Complex adaptive system

## Abstract

Understanding how individual scientists build a personal portfolio of research is key to understanding outcomes on the level of scientific fields, institutions, and systems. We lack the scientometric and statistical instruments to examine the development over time of the involvement of researchers in different problem areas. In this paper we present a scientometric method to map, measure, and compare the entire corpus of individual scientists. We use this method to analyse the search strategies of 43 condensed matter physicists along their academic lifecycle. We formulate six propositions that summarise our theoretical expectations and are empirically testable: (1) a scientist’s work consists of multiple finite research trails; (2) a scientist will work in several parallel research trails; (3) a scientist’s role in research trail selection changes along the lifecycle; (4) a scientist’s portfolio will converge before it diverges; (5) the rise and fall of research trails is associated with career changes; and (6) the rise and fall of research trails is associated with the potential for reputational gain. Four propositions are confirmed, the fifth is rejected, and the sixth could not be confirmed or rejected. In combination, the results of the four confirmed propositions reveal specific search strategies along the academic lifecycle. In the PhD phase scientists work in one problem area that is often unconnected to the later portfolio. The postdoctoral phase is where scientists diversify their portfolio and their social network, entering various problem areas and abandoning low-yielding ones. A professor has a much more stable portfolio, leading the work of PhDs and postdoctoral researchers. We present an agenda for future research and discuss theoretical and policy implications.

## Introduction

There is continuing interest in the micro-level dynamics of science, particularly to better understand how policy affects the science system. One of the most important problems in science policy concerns the definition and realisation of scientific priorities (Dasgupta and Maskin 1987). Governments, funding councils, universities, and individual researchers are continually searching for the most promising and dynamic areas. They use a wide range of instruments to shift resources and align research agendas, including funding opportunities, research coordination and power (Lepori 2011) as well as incentives and inspiration (Verbree et al. 2012b).

A major challenge of developing institutional and national research priorities is that science is a complex adaptive social system (Wagner and Leydesdorff 2005). Bound by the rules and structures laid down by government and the scientific community, national and institutional portfolios emerge from the simple rules that drive the behaviour of individual scientists and research groups. The results can be unexpected, even counterintuitive. Understanding how individual scientists apply those rules to build a personal portfolio of research is key to understanding outcomes on the level of scientific fields, institutions, and systems. How do scientists develop their research agenda? What is their search strategy? And where policy instruments are used to align or shift research agendas, how can we tell if they have successfully changed the behaviour of individual scientists?

Few exceptions notwithstanding (Laudel and Gläser 2008; Zuckerman and Cole 1994), there has been little attention for the way in which scientists develop a research portfolio in the course of their career. The most important obstacle is that we lack the scientometric and statistical instruments to examine the development over time of the involvement of researchers in different problem areas. In this paper we present a novel scientometric method to map, measure, and compare the lifetime corpus of individual scientists. We provide proof of concept by using this method to analyse the search strategies of 43 condensed matter physicists along their academic lifecycle.

In “Conceptual framework” we develop six propositions. The scientometric methods are developed in “Methods and data”. We then test our propositions in “Results” by applying the methods to data on the lifetime publications of 43 physicists. We analyse the results in “Analysis”. In “Conclusions and discussion” we summarise the main conclusions and discuss the implications.

## Conceptual framework

The dynamics of the scientific search for new knowledge have attracted the interest of a wide variety of scholars for over half a century. Empirically, the main emphasis has consistently been on the development of researcher productivity along an academic career in relation to the main incentives (Stephan and Levin 1992). A similar line of research concerns star scientists, specifically what distinguishes stars from ordinary scientists (e.g. Zucker and Darby 1996; Zuckerman 1992) and how to identify them in terms of output and productivity in relation to age (Costas et al. 2010) and role (Bayer and Smart 1991). Lifecycles have been studied to examine changes over time in the productivity of researchers (Carayol and Matt 2004, 2006; Falagas et al. 2008; Levin and Stephan, 1991; Reskin 1977) and the activity profile of institutes (Braam and van den Besselaar 2010).

Research productivity deals with aggregate output, while search suggests the possibility of exploring multiple topics of research. An individual scientist’s portfolio reflects his curiosity and the opportunities with which he is presented, such as new talent in his group, new funding opportunities, emergent research themes, or simple serendipity. Scientific portfolio management is all about spreading risks, maximizing reputational gains, and satisfying personal (intrinsic and extrinsic) motivations.

### The reward system of science

Since Merton’s more philosophical work on the reward system of science (Merton 1957, 1970), scholarly behaviour has been explained as a search for priority with reputation as a reward. Scientists strive to be the first to find an original result, while their peers in the field review and validate their work, thus assigning value and giving recognition. The reward system of science was further elaborated and empirically tested from a sociological perspective (e.g. Cole 1970; Cole and Cole 1967; Reskin 1979). Priority seeking and reputation were also at the heart of the new economics of science that arose in the 1990s (Dasgupta and David 1994; Stephan 1996; Stewart 1995). It is also now understood that scientists respond to a range of different, sometimes competing incentives, including the need to search for priority and establish a reputation, external demand for the results of a project, their own interest or curiosity, and—to a lesser extent—extrinsic rewards such as prizes, honours, and salary (e.g. Calderini et al. 2007; Verbree et al. 2012a).

Zuckerman and Cole (1994) show how the reward systems functions. They used interviews to find out if eminent scientists use different research strategies than ordinary scientists, which might account for their higher performance in numbers of publications and citations. The researchers Zuckerman and Cole interviewed selected problems based on three criteria: (1) how important they believe the problem to be as well as how their peers will respond when it is solved, (2) how easy or difficult it will be to solve the problem, and (3) how long it will probably take to get results. These criteria were then set off against the degree of competition around the problem.

“Are a good many others working on the problem and is the competition apt to be stiff? By and large, these established scientists say they consider it a waste of time to work on problems actively being pursued by others. […] Although the presence of competition may deter scientists from taking up a particular problem, it is apparently not sufficient to prevent them from doing so if the problem involved is judged of prime scientific significance—and if they think they can solve it first.” (Zuckerman and Cole, 1994, pp. 396–397)

According to Zuckerman and Cole, “eminent” scientists are more willing to engage the competition, while “rank-and-file” scientists prefer to avoid problems that others are working on. The complexity of the problem also matters. More complex problems take more time to solve. Such niches will be less crowded, but the potential reputational gains are higher. They will tend to attract fewer but more eminent researchers, thus raising the risk of being scooped.

Hagstrom (1974) used surveys to measure similar considerations twenty years earlier. He shows that the motivational drivers proposed by Merton and others actually work. Problem selection is associated with competitive intensity and personal ability to compete wherein the perceived intensity of competition varies by discipline and age. Hagstrom also noted possible perverse effects of intense competition, such as questionable conduct (Anderson et al. 2007) and an increase in secrecy among researchers (Hong and Walsh 2009). He predicted that the nature of scientific competition might change if the social organisation of science changed, for example in response to the rise of big science. Several authors witness such an increase in competition. For example, Rauber and Ursprung (2008) examined the productivity of different age cohorts of German economists over time. Their results show the increasing competitiveness of academia: younger cohorts are far more productive than older cohorts. Hessels (2010) shows that competition is becoming more intense. Within science, there is an ever stronger pressure to publish or perish. At the same time, there is an increasing call for science to produce socially (or economically) relevant knowledge.

### Community

The literature on the reward system of science shows that problem choice is the main instrument of competition between an individual scientist and his peers. Problem choice is driven by the possibility of gaining reputation. Yet, reputational gains require a community of peers who work on the same or similar problems and can recognize achievement.

The work of Zuckerman, Cole, and Hagstrom suggests that scientists select problems based on their private perception of the trade-off between community size and potential marginal reputational gains. It is relatively easier to be the first to find a result in a very small niche, but there will be fewer peers to recognise the achievement. In a very large or crowded niche, achieving priority is much more difficult, but the potential gains may be much higher than in a small niche. The search for a niche and the need for a community that grants a reputation are consequently at odds. Perceptions of the trade-off will change over time, owing to the accumulation of reputation, changes in academic status, and experience. It may be easier for a reputable scholar than a rank-and-file scientist to produce a high-impact contribution in a crowded field and the same may be true for publishing work on an entirely new, self-defined problem. Highly complex problems attract fewer but eminent researchers, while rank-and-file researchers crowd specialties of less complex problems (Zuckerman and Cole 1994).

The decision space of scientists is bounded by the requirements of their global community of peers and by local conditions and opportunities. Whitley (1974) refers to the degree of cognitive institutionalisation of scientific fields, which depends on “the degree of consensus and clarity of formulation, criteria of problem relevance, definition and acceptability of solutions as well as the appropriate techniques used and instrumentation.” (Whitley 1974, p. 72) Where the quest for priority—i.e. originality—creates divergence and task uncertainty, the need for reputational gains forces scientists to conform to a community of peers who can validate and replicate their work (Whitley 2000). This community structures the search for new problems. In other words, for a scientist to be able to compete he has to have competitors with whom he has to reach a level of consensus on the basic premises of the problem area.

The interaction between a scientist and his community can be seen as a way to gather the resources necessary for doing research on a particular problem (cf. Pfeffer and Salancik 1978). By collaborating with their peers, scientists gain access to crucial resources. High academic reputation, specific expertise, and access to facilities, equipment, and data are the fuel for preferential attachment (e.g. Birnholtz 2007; Bozeman and Corley 2004; Melin 2000; van Rijnsoever et al. 2008). We can also take a more sociological view. In the words of Lave and Wenger (1991), scientists form communities of practice around problem areas, in specialties, and in fields. Scientists learn from each other by sharing knowledge, for example at conferences or through collaboration. The communities they form have a formal dimension—think, for example, of academic associations—but more often they are self-organising or emergent (e.g. Brown and Duguid 1991). From this perspective, it is interesting to note the rise of team science around some of the hardest problems in science (Stokols et al. 2008).

Problem choice is one of the ways in which individual scientists strategically position themselves in a wider environment. They develop their own research topics that latch onto problem areas defined by their community or by society at large as expressed in public debates or in funding opportunities. Problem choice, the accumulation of reputation, community development, and the collection of resources are dynamically interrelated.

The economic model in which behaviour is driven by individual rules and preferences in interaction with an outside environment provides a good understanding of problem choice in science. It is important to keep in mind that models are a simplification of reality. Most scientists are motivated by more than possible reputational gains but are also intrinsically motivated. Models cannot capture all dimensions of their behaviour. This is particularly true for curiosity, creativity, and serendipity, that introduce a degree of randomness in problem choice. However, even though each individual scientist will have a private heuristic, at system level or across large populations of researchers the model will hold.

### Search strategies

The aim of our analysis is to characterise the search strategies of scientists. “Search” denotes the process by which an individual scientist identifies, enters, develops, and exits a problem area and its associated community of peers. We refer to a scientist’s activity in one problem area as a research trail. “Strategy” refers to the scientist’s strategic positioning in a competitive environment and presumes a degree of planning, coherence and consistency to problem choice over time. We expect search strategies to evolve along the academic lifecycle. The search process and the strategy behind it are dynamic and interrelated, each developing in response to changes in status and position, the availability of resources and access to social networks, the constraints imposed by prior work, and unexpected findings and opportunities. We can see an individual scientist’s search strategy as the way in which he negotiates his way through Bonaccorsi’s search regimes (2008).

### Propositions

In this section we develop a number of propositions with regard to the social and cognitive dynamics in the work of individual scientists that our method should be able to measure.

#### **Proposition 1**

*A scientist’s work consists of multiple finite research trails*

Scientists develop along an academic lifecycle. As they age, scientists gain experience, develop a set of skills and achieve reputational gains, which affects their ability to gain access to critical resources for the next problem (Kyvik and Olsen 2008). And problems can be solved, if not by the researcher himself then by his peers.

#### **Proposition 2**

*A scientist will work in several parallel research trails*

Scientists can be active in different niches, each with its specific consensus on the most important problems and the state-of-the-art of data, resources, standards and criteria. If task uncertainty is high then consensus among peers is low and scientists can earn recognition for a more diverse set of problems (Whitley 2000). This implies that a scientist may work for different communities of peers, using different funding sources, and working with different networks of collaborators (Zuckerman and Cole 1994, pp. 398–399). Flexibility in problem choice does depend on access to resources, including a team of researchers (and their inherent knowledge and skillsets), funding, facilities, and data.

#### **Proposition 3**

*A scientist’s role in research trail selection changes along the lifecycle*

Scientists progress up the academic hierarchy from PhD to postdoc to professor. This progression changes the role they play in problem choice. In addition, the lifecycles of individual researchers overlap and they interact while at different stages of their individual lifecycles (Dietz et al. 2000). A good example is that of the PhD student and his supervisor. At the start of a scientist’s career, his supervisor may assign him a problem to solve, while as professor he assigns problems to his PhDs. Over time, individual autonomy with respect to problem choice will increase and involvement in problem areas will change from first-hand involvement in one narrow problem area to a supervisory role in a range of problem areas. As research leaders, individual scholars supervise, inspire, and manage a collective of researchers at varying levels of academic development.

#### **Proposition 4**

*The start and end of research trails is associated with career changes*

The behaviour of scientists changes over time. Scholars rise in the academic hierarchy, move between institutions, and develop a social network. Verbree et al. (2012a, b) show that the behaviour of medical research group leaders varies according to their age, the phase of their lifecycle (especially when they near retirement), and the dominant incentives in the science system during their PhD phase. As their status changes—from PhD student to postdoctoral research to full professor—so does their role in agenda setting. A higher function provides new capabilities. Moving to a different institute gives access to new expertise, better facilities and support, a different environment. Academic careers are not uniform. Dietz and Bozeman (2005) show that there are different paths of academic advancement and that the nature of careers has changed over time. They also find that job changes boost researcher productivity.

#### **Proposition 5**

*The start and end of research trails is associated with the potential for reputational gain*

Scientists enter new niches in the hope of accumulating additional reputation. The implication is that if the niche does not deliver, they will abandon it. Behaviour may change along the lifecycle. As Hagstrom (1974) put it: “the marginal value of each discovery is greater for younger men”. Also, as a scientist accumulates reputation, the probability that other scientists will want to collaborate (and co-author) for work in the same area and in adjoining areas rises (Melin 2000). Activity in a problem area may become self-sustaining.

#### **Proposition 6**

*A scientist’s portfolio will converge before it diverges*

Problem choice is a non-random process. Each decision is linked to the previous and we can expect most research trails to be connected. After all, a scientist builds on his accumulated stock of expertise, reputation, network relations, and resources. A succession of research trails creates path dependency. We expect to find that after a scientist finds his core niche and discovers his reputational blockbuster, his portfolio will tend to converge. Only later in his career, when the potential for marginal reputational gains as well as the risk of entering new niches goes down, will divergence occur.

## Methods and data

In this section we explain how we map an individual scientist’s portfolio over time and which data were used to do the mapping. In the “Results”, the method will be applied to empirically test the propositions.

### Method

There are several ways to map the structure of scientific fields. Boyack and Klavans (2010, 2009) use co-citation analysis to map the grand structure of science. They identify current paradigms and their relative position in the entire body of scientific output. A similar but different method is bibliographic coupling (e.g. Jarneving 2007). Science overlay maps allow researchers to reveal the disciplinary orientation of a researcher, country, institution or field by mapping the subject areas in the relevant body of output on the journal structure of science (Rafols et al. 2010). Such maps show structures that emerge from underlying social and cognitive dynamics that have been studied in the literature since the 1950s. We know that the dynamics of science can be traced to the behaviour of individual scientists. Yet, we lack the methods to map and measure those dynamics.

To meet the requirements of our analysis, we need to adjust extant methods in two ways. First, we must find a way to map paradigms or clusters in a set of publications over time, showing both the development of each individual cluster and the degree of similarity between clusters. Second, our method must capture the search for priority as well as the need for a community. What we want to measure is thematic selection and strategic positioning: selection in context. This means that we are looking for two dimensions. Along the first dimension an individual researcher demarcates his discrete epistemic niche. This describes what he or she actually researches. Along the second dimension a researcher connects to a specific community of peers. These are the people who study the same problem and who review, validate, and replicate their work.

Van den Besselaar and Heimeriks (2006) have developed a method that measures along these two dimensions. They map the structure of a field by measuring the similarity between publications in terms of shared combinations of title words and cited references. Title words catch the first dimension by describing the contents of a publication. Cited references capture the second dimension. Authors who refer to the same body of literature work in the same research tradition. They form a community of peers. The result may be considered a proxy for the epistemic culture to which a researcher belongs. Title words and cited references capture the researcher’s contribution to ‘what we know’, the “signature of the knowledge claim” (Lucio-Arias and Leydesdorff 2009), while his reputation is decided by a collective of peers in the same community (Knorr-Cetina 1999).

We map the structure of an individual researcher’s lifetime portfolio using title word-cited reference combinations to calculate the similarity between publications. The SAINT toolkit (Somers 2009) was used to transform raw data into networks of similar papers for analysis and visualisation. The ISI parser turns raw Web of Science data into a relational database. This allows the user to examine any possible combination of data. The Word Splitter parses titles and abstracts into individual words, providing a stemmed version of each word using the Porter stemming algorithm (Van Rijsbergen et al. 1980) and removing user-defined stopwords. From the relational database, we extract combinations of stemmed title words and cited references. For each pair of publications A and B, the Tanimoto coefficient (a derivative of the Jaccard similarity coefficient) τ is calculated:

$$ \tau ( {\text{A,B)}} = \frac{{N_{\text{AB}} }}{{ (N_{\text{A}} + N_{\text{B}} - N_{\text{AB}} )}} $$

where *N* A is the count of word-reference combination tokens in A, *N* B is the count of tokens in B and *N* AB is the count of tokens shared between A and B. This gives us the basic data needed to construct a network consisting of a set of publications (the nodes) and a similarity between each pair of publications (the edges).

The Community Detection Tool within SAINT uses the community detection algorithm of Blondel et al. (2008) to demarcate clusters of highly similar publications within the network.^1^ Blondel et al.’s method identifies sets of highly interconnected nodes within large networks producing a community structure with high modularity (i.e. high density of links within communities and low density of links between communities). Their algorithm has three distinct advantages: (1) it is a multi-level algorithm that shows the hierarchical structure of the network and allows analysis of communities at different levels of aggregation; (2) it is able to detect very small communities; and (3) it resolves very rapidly across large networks. In our analysis, communities represent specialties within the academic corpus of researchers. Some publications have no similarities to other publications in a corpus. The community detection algorithm isolates these into single-node communities. These papers have been ignored.

We focus only on citable documents, that is, articles, conference proceedings, letters, notes, and reviews.^2^ Citeable documents are the foundations of an academic career: it is through citation that we can measure the marginal reputational gain that a publication produces. Where we map portfolios and scale the size of individual publications and where we analyse reputational gains, we will use average annual citations received until the time when we downloaded the data (March–May 2011). Total citations will tend to overestimate the impact of older publications. This is of course an oversimplication of the problem, as can be read in numerous articles about citation ageing (Bouabid 2011).

CV data are used to differentiate between the PhD, postdoctoral and professorial phases of an academic career as well as to identify the major career moments. Major career moments include changes in position (e.g. postdoctoral researcher, associate professor) and moving between institutions; visiting scholarships, honorary chairs and other similar positions are ignored. Associating CV dates with publication dates requires an adjustment for the lag between submitting a paper—the final stage of doing the actual research—and its publication in a journal or conference proceeding. It takes time to set up a project, acquire funding, hire researchers, do the work, write papers, and get them published. For each analysis we have tested the effects of different lags. In this paper, we present the results for a 2-year publication lag.

**Fig. 1:**
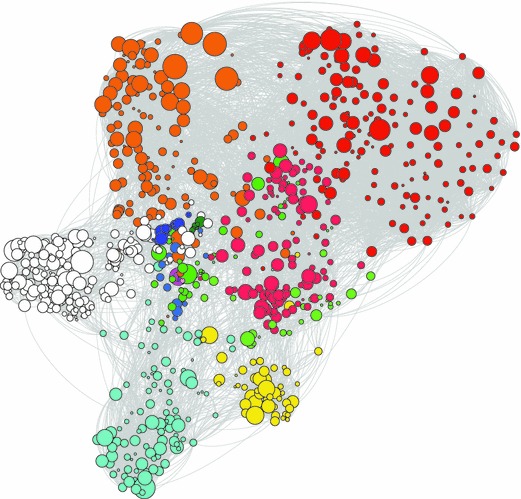
Map of Zachary Fisk’s publications in two-dimensional space using a force-directed algorithmNote: Similarity in terms of title word-cited reference combinations. Size of the nodes indicate average annual number of citations from date publication to 2011. *Colours* indicate clusters (colour figure online)

The challenge is to show how one individual scientist has developed his portfolio over time, possibly working in several problem areas simultaneously, some of which are similar and others dissimilar. Mapping the lifetime corpus of an individual scientist in two-dimensional space produces visualisations that tend to look like those in Fig. 1. Each node is a publication. The size of a node indicates the annual average number of citations received from the date of publication until the moment of downloading, thus normalising for the fact that older publications have had more time to accumulate citations. The colours of the nodes represent the different problem areas in the scientist’s corpus. Each edge represent a similarity and has a weight equal to the degree of similarity (the Tanimoto coefficient). The nodes are positioned using the Force Atlas layout, a variant of the Fruchterman–Reingold force-directed algorithm embedded in Gephi (Bastian et al. 2009). Nodes that are highly similar are clustered close together; nodes with low similarity or no similarity are positioned farther apart.

**Fig. 2:**
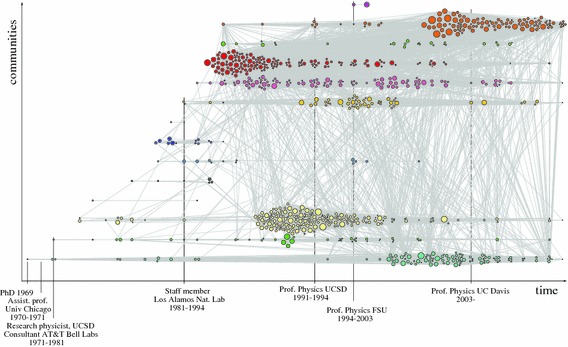
Clusters in the corpus of Zachary Fisk mapped over time and linked to CV data

Figure 2 presents a novel method for mapping a corpus of publications. The information in this figure is identical to Fig. 1. What is new is that we have arranged the publications along two axes, that of time on the *x* axis and problem areas on the *y* axis. Longitude is defined as [(year of publication) − (year of first documented publication in the dataset)/(range in years) × 360] − 180. Latitude is defined as [(community number)/(total number of communities) × 180] − 90. The nodes were positioned with the GeoLayout in Gephi, using an equirectangular projection. Since this positions every publication in one problem area and one year on exactly the same location, we use the Noverlap function to force the nodes to be shown side by side. This method allows us to view over time the emergence and development of activity in different problem areas, showing potential overlaps and interrelations.

Three caveats must be made.

1. *Self-citation* Self-citations may artificially imbue coherence to the lifetime corpus of one individual. In the first publication, the researcher cannot refer to own publications. Later in his career he may use self-citation to raise the impact—and hence reputation—of his work (van Raan 2008b). Similarly, when he enters a new niche of limited size—either self-defined or following a few prior publications—there are few other publications he can refer to. This is precisely where we find the difference between mapping problem areas in a wider community and mapping problem choice by an individual scientist who strategically positions himself in his global and local environments. It is the latter that we want to map and for this reason self-citations are included. An interesting approach, similar to ours, is presented by Hellsten et al. (2007) who use self-citations to trace how a scientist moves between research topics. The difference is that a method based on self-citations seems less accurate for early-career researchers.
2. *Aggregation* Our method maps problem choice by individuals. This represents the lowest level in Whitley’s (1974) scheme of aggregation in which a field consists of specialties that consist of research areas that contain problem situations. The research trails that we identify represent the individual scientist’s selection of problem areas that relate to research areas at higher levels of aggregation. Klavans and Boyack (2011) find that global maps of research fields are more accurate than local maps, which seems to argue in favour of mapping individual portfolios in their global context. However, our method provides a highly fine-grained and individual perspective of the way in which a scientist develops a portfolio. In addition, it also works on very small data sets.
3. *Who decides and how* Our method presumes that problem choice is a decision of the individual scientist. It consequently disregards group publication strategies, hyperauthorship (e.g. in particle physics), and the rise of team science. Also, there will be disciplinary or community differences in publication and citation cultures (van Raan 2008a; Wouters 1999; Zuckerman 1987) and a scholar’s role in problem selection is likely to change over time.

### Data

A sample of individual scientists was constructed to develop the methods and extract statistical measurements. We focus on a single specialty, namely condensed matter physics, and start our search with distinguished scholars who work or have worked at high-magnetic field labs in Tallahassee, Nijmegen, and Dresden that serve as focal points in the field. In addition, we extracted the top-25 American, Dutch, and German authors from ten important journals in condensed matter physics (Physical Review B, Journal of Physics-Condensed Matter, Thin Solid Films, Physica B-Condensed Matter, Advanced Materials, Applied Surface Science, Surface Science, Journal of Magnetism and Magnetic Materials, Journal of Nanoscience and Nanotechnology, and Physica Status Solidi B-Basic Solid State Physics). From the list of potential candidates, we first selected those with a long and distinguished career and good CV data. In addition, some of the selected physicists have had a shorter career or have achieved a less exalted status in their field than others. The result is a sample of 43 condensed matter physicists.

The lifetime publications of the 43 physicists were downloaded from Thomson Reuters Web of Science.^3^ The publications retrieved from the Web of Science were manually checked to ensure that they belong to the work of the selected physicists. This check involved a comparison with the scientist’s curriculum vitae, an analysis of the subject areas of the retrieved papers, and a comparison with lists of publications on personal websites and in CVs. The corpus is not necessarily complete. The Web of Science does not include every single academic publication. For example, some of the early publications of Russian physicists—for their PhD and the Doctor of Science theses—have only been published in Russian and may not be included in the dataset. Also, the Web of Science has expanded over time, so coverage today is better than it used to be at the beginning of the careers of our subjects. However, overall coverage is sufficient for the purposes of our analysis.

## Results

This section presents the results of the empirical tests of the six propositions.

### **Proposition 1**

*A scientist’s work consists of multiple finite research trails*

**Fig. 3:**
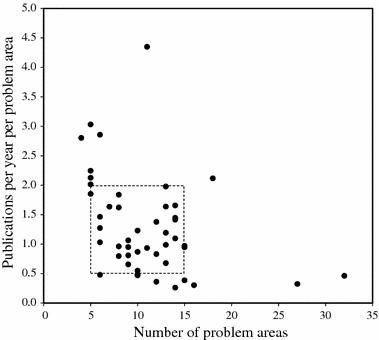
Number of research trails and the average intensity of activity per research trails

Together, the 43 physicists have published 18,235 publications and worked on 459 problem areas during their career. On average, in the course of their academic career the physicists in our sample have been (or still are) active in 11 different problem areas (a minimum of 4 and a maximum of 32), producing about 43 publications per problem area and 1.3 publications per area per year. A closer look at the distribution within our sample (Fig. 3) shows that the career output of most researchers clusters into 5–15 problem areas with between 0.5 and 2 papers per problem area per year.

On average, they remain active in a problem area for just over 13 years or 39 % of their career length. Among the 459 individual problem areas about 41 % lasted five years or less and about 26 % covers all or most of a career. In other words, research trails are finite and scientists will work in multiple trails in the course of their career. Therefore, proposition 1 is confirmed.

### **Proposition 2**

*A scientist will work in several parallel research trails*

Figure 2 shows how an individual scientist tackles different problem areas in a specific order. After obtaining their PhD, they expanded to other areas and, significantly, began to work in several problem areas at the same time. Their work converged on a core of highly similar research trails in which they worked for many years and that constitute the basis of their current reputation. Considering the total number of problem areas which the physicists in our sample have worked on during their entire career, it is quite likely that they worked on two or more problem areas at the same time, much like the example in Fig. 2.

**Fig. 4:**
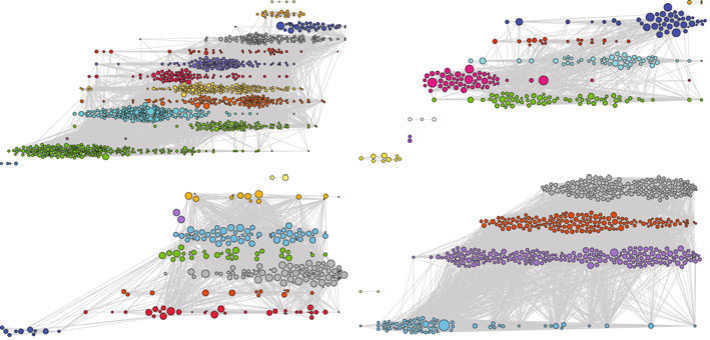
Visualisation of the lifetime corpus of four condensed matter physicists with time on the *x* axis and problem areas on the *y* axis

Figure 4 shows the portfolios of four condensed matter physicists from our sample. The portfolios seem very different. They consist of different mixtures of short and disconnected research trails intermingled with very long and active trails. Within trails we can see bursts of activity as well as periods of intermittent activity. Some portfolios show more focus while others are more diverse. The underlying statistical patterns and sociological drivers may, however, be the same.

The number of problem areas in which a scientist is active at any one time can be considered a measure for the diversity of his portfolio. How diverse is the portfolio of our condensed matter physicists in different phases of their academic lifecycle? We distinguish between three phases: the PhD phase, the postdoctoral phase, and the professorial phase. The phases were demarcated using CV data.

**Fig. 5:**
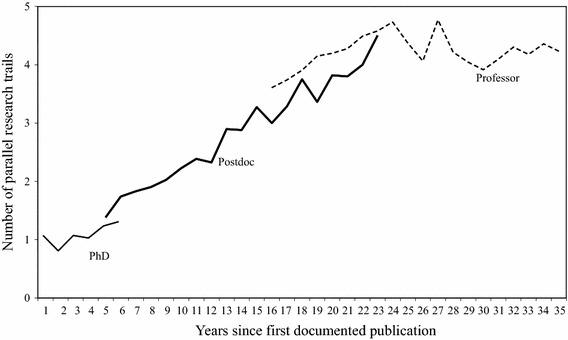
Average number of parallel research trails for the PhD, postdoctoral and professorial phases of the academic lifecycle (assuming a publication lag of two years)

Figure 5 presents the average number of parallel research trails in the three phases of the academic lifecycle, using years with at least ten observations. The beginnings of the postdoctoral and professorial phases were placed in the year when the average scientist in the sample entered this particular phase. There is an overlap between phases as not all scientists began or ended a particular phase in the same year of their career. The figure shows that PhDs, as expected, tend to work in one problem area only. This is followed by an expansion of the portfolio during the postdoctoral period, which appears to be the period in which our scientists search for a core specialisation. As professors, the number of parallel problem areas is steady at around 4. Following this, we state that proposition 2 is confirmed.

### **Proposition 3**

*A scientist’s role in research trail selection changes along the lifecycle*

**Fig. 6:**
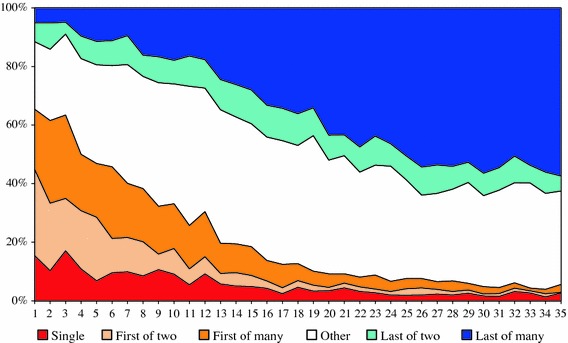
Percentage share of different author positions from first documented publication

**Fig. 7:**
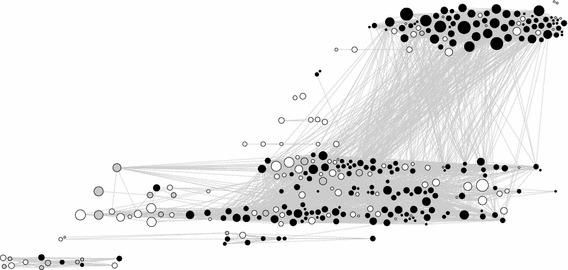
Visual representation of author positions over time in different problem areas for T. Rasing Note: *gray* = first author; *black* = last author; *white* = other author positions

Figure 6 shows the percentage share of each author position in the annual output of the sample of scientists since the first documented publication. The figure shows that in the first few years of their career the sample physicists were first or only author on approximately two-thirds of their publications. During the first 10–15 years this percentage fell sharply until, by the 15th year, only about 20 % of papers was written as first or only author. This decline was compensated for at first by a simultaneous increase in the number of last and other author positions and, later in their career, by a further increase in the percentage share of last author positions. After about 30 years of publishing, the sample physicists were last author on 50–60 % of their publications. Figure 7 shows how one scientist progressed in the author list throughout his career.

**Fig. 8:**
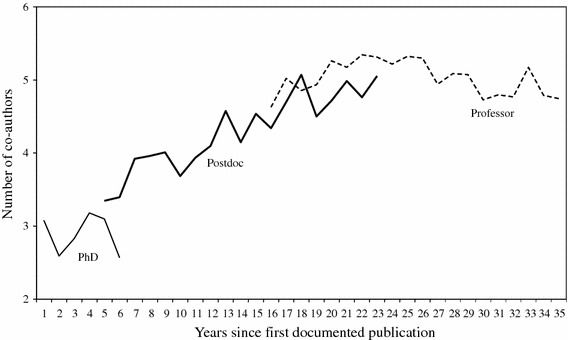
Average number of co-authors in the PhD, postdoctoral and professorial phases of the academic lifecycle (assuming a publication lag of two years)

Table 1 presents the percentage share of each author position in total output per phase. The results confirm that the majority of papers (56.4 %) in the PhD phase was written as first or single author. In the postdoctoral phase, the number of papers written in other or last author positions increased rapidly. The increase in other author positions may suggest that in addition to an increase in output and a diversification of problem areas, scientists expand their coauthor networks during their postdoctoral phase. This is evident from the development of the average number of coauthors per paper in the three career phases (Fig. 8). Perhaps, this is where they become active in interinstitutional collaborations and decisions on problem choice are taken collectively rather than individually. In the professorial phase, we observe a relative decrease in other author positions and a strong increase in last author positions. The shift in author positions provides a good indication for the changing role of scientists in problem choice in the course of their career. Hence, proposition 3 is confirmed.

**Table 1:** Percentage of publications written in first, other or last author positions in three phases of the academic lifecycle

Author position	PhD	Postdoc	Professor
First	56.4	22.8	19.7
Other	28.9	39.6	29.4
Last	14.8	37.6	50.8
Total	100	100	100

### **Proposition 4**

*The start and end of research trails is associated with career changes*

Is there an association between key events in a scientist’s career and the rise and fall of research trails? We have compared the timing and duration of career phases (PhD, postdoctoral, professorial) and career moments (key events such as promotion or moving to another institute or country) with the timing of the start and end of research trails to find out if such an association exists.

First, we have examined in which phases of an academic career research trails start and end. Table 2 shows that the postdoctoral phase is the most active in terms of the net increase in research trails. As PhD students they begin with one trail and by the time they graduate most will have explored one additional problem area. Postdoctoral researchers start working in 4 or 5 different problem areas and only end an average of 1.7 research trails. This matches our earlier analysis of parallel trails and the expansion of a scientist’s portfolio in the postdoctoral period. We also find a match in the professorial phase when the scientists in our sample started about as many trails as they abandoned. The data in Table 2 show that there is an association between career dynamics and problem choice. From this, the question arises if it is the career *phase* or the career *moment* that matters.

**Table 2:** Number of research trails that started or ended in different phases

	Started		Ended	
	Average number	Average percentage (%)	Average number	Average percentage
PhD phase	2.0	24.9	0.4	4.2
Postdoctoral phase	4.6	42.2	1.7	17.2
–earlier moments in the postdoctoral phase	2.5	23.4	0.6	6.0
–final moment before becoming professor	2.1	18.8	1.1	11.2
Professorial phase	4.0	32.9	4.4	37.0
Not abandoned			4.1	41.7

Note: Assuming a two-year publication lag. The percentages are an average of the percentage distribution of research trails of the individual scientists and do not relate (directly) to the average numbers in the table

Table 3 shows that the scientists in our sample remain in one position or location longer as they progress up the academic hierarchy. The average postdoctoral career moment lasts 3.9 years; the final unfinished professorial moment 12.2 years. New research trails are started on average every 2.5 years, which is so close in length to a postdoctoral career moment that it is hard to tell if the association is between phases or moments.

**Table 3:** Time between career moments compared to the time between the start of new research trails (years)

	Average length	95 % interval	Standard deviation	N
Postdoctoral moment	3.9	3.2–4.7	3.1	71
Final moment before becoming professor	5.3	4.4–6.2	2.9	37
Career moments after having become professor	6.2	4.9–7.6	5.2	55
Final unfinished moment	12.2	10.3–14.0	6.1	42
Time between the start of research trails	2.5	2.3–2.8	3.0	467

We have examined the percentage of research trails that were started in the first 4 years after a career moment in the postdoctoral and professor phases. By the fourth year, 62 % of all research trails initiated during the postdoctoral phase had started compared to 51 % of all trails initiated during the professorial phase. For abandoned trails the percentages are 58 and 41 % respectively (assuming a two-year publication lag). When we normalise these percentages for the average length of a career moment, the results speak in favour of professors. The average length of a professorial career moment is 1.6 times longer than the average postdoctoral moment (6.2 vs. 3.9), while the probability of trails starting or ending in the 4 years after a career moment is only 1.2 and 1.4 times higher for postdoctoral researchers. In other words, professors have more time to start or abandon trails but they are more likely to do so in the first 4 years after a career moment than postdoctoral researchers.

To complicate matters further, there may in some cases be an association between career changes and the continuation of research trails started in an earlier phase. Work that earned someone a postdoctoral position or professorship may well be continued after an appointment. The association between career moments and problem choice may not relate to the start or end of a research trail.

In summary, the data suggest that there is an association between career *phases* and portfolio development. However, we cannot prove that there is an association between career *moments* and the start or end of research trails. From this, proposition 4 is neither confirmed nor rejected.

### **Proposition 5**

*The start and end of research trails is associated with the potential for reputational gain*

Citations received can be considered a good proxy for recognition, that is, the contribution of a publication to a scientist’s reputation. Scientists enter problem areas because they expect to achieve marginal reputational gains; they abandon them when the expectation is not met or the potential gains in terms of reputation and recognition are depleted. Following this, are the ends of trails associated with citations received as the main indicator of reputation? If so, then by association the starts of trails are also associated with citations received.

We distinguish three types of research trails: (1) short trails, defined as trails that (a) exist for no more than 5 contiguous years or (b) represent no more than a total of 5 years of activity; (2) lifetime trails with activity throughout most of a career; and (3) intermediate trails that encompass more than 5 years of (contiguous) activity but less than an entire career. Among the research trails of the 43 condensed matter physicists there are:

- 189 short trails that represent no more than 5 total or contiguous years of activity, lasting an average of 3.7 years, of which 152 are short trails that exist for no more than 5 years and last an average of 2.2 years;
- 121 lifetime trails that last an average of 26.9 years;
- 149 intermediate trails that last an average of 14.6 years.

In other words, of all the research trails in our sample 41.2 % is short, 26.4 % lifetime, and 32.5 % of intermediate duration.

**Fig. 9:**
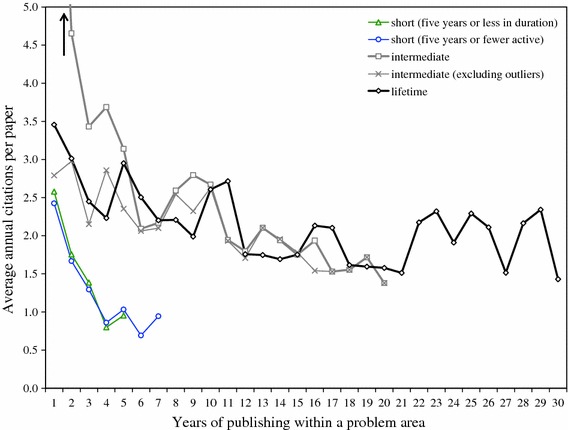
Comparison of average annual citations per publication in short, lifetime and intermediate research trails

The development of average annual citations per publication clearly shows the difference between short research trails and longer trails (Fig. 9). Short trails start from around 2.5 citations per year and drop below 1 in the fourth year. Lifetime trails start from around 3.5 citations per year and gradually decline to just under 2. Initial inspection of intermediate trails shows average annual citations starting at just under ten on average. This turned out to be due to eight very specific trails of seven scientists, including some of the most highly cited papers in this field (e.g. Geim’s work on graphene, which has received thousands of citations since 2005). After adjusting for these outliers, the development of average annual citations for trails of intermediate length was almost identical to that of the lifetime trails.

Short trails apparently fail to deliver the expected marginal reputational gains, as evidenced by the short, sharp drops in average annual citations, and were consequently abandoned. The reasoning for these drops are not evident from the data, but Bornmann and Daniel (2010) provide additional support for the hypothesis that the speed with which citations are received is an indication of reputational gains. Articles accepted for publication in a highly reputable chemistry journal received their first citations faster than rejected articles that were published elsewhere (see also van Dalen and Henkens 2005). Similarly, Drucker and Goldstein (2007) show that early citations are a good predictor for lifetime citations. Conversely, reputational gains cannot explain why intermediate trails are abandoned and lifetime trails are not. We can, however, speculate that this may be due to developmental hurdles or oversaturation of the field. To test for such phenomena, future analyses will have to compare the individual’s activity with worldwide production in a problem area to ascertain if the decrease in activity in the area is universal amongst all other researchers in the field or specific to the researcher in question. Without more detailed evidence we can only state for our purposes that proposition 5 is confirmed only for short research trails.

### **Proposition 6**

*A scientist’s portfolio will converge before it diverges*

Bonaccorsi (2008) examines the dynamic properties of the scientific search process at the meso level of scientific fields. He characterises a search regime based on three properties: the rate of growth, the degree of diversity (search regimes can be convergent or divergent) and complementarities between the required resources. It has proven very difficult to establish empirically whether or not fields are convergent or divergent. We can also use Bonaccorsi’s model to characterise the search regime of individual scientists. By mapping the lifetime development of their portfolio, we can empirically test for convergence and divergence at the individual level.

**Fig. 10:**
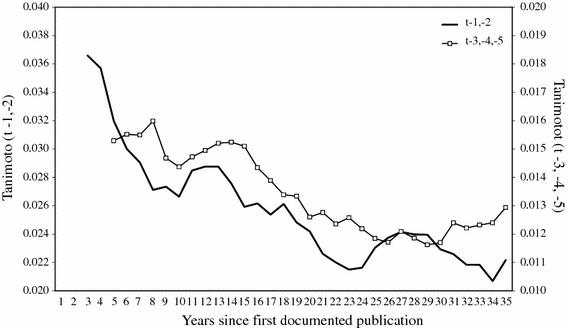
Similarity between an author’s papers published in year t and his papers published 1 or 2 years or 3, 4 or 5 years previously (Tanimoto coefficients; three-yearly moving averages) Note: One scientist’s data were removed from the sample due to extreme outliers

Figure 10 shows the degree of similarity between papers published in year *t* to each scientist’s own papers published in preceding years. We have tested for the two preceding years (t-1 and t-2) and for the 3 years before that (t-3, t-4 and t-5). The figure shows that similarity declines almost continuously in the first 20 years of publishing. Nearer the end of the average career we find a mild increase in similarity. The later increase in similarity occurs in the professorial phase.

The decline in similarity after the PhD phase is probably related the expansion of portfolios observed in the postdoctoral phase. It is in the nature of the method used to identify problem areas—specifically the fact that Blondel et al.’s algorithm optimises for modularity—that similarity is high within areas and low between areas. An increase in the number of parallel problem areas exerts downward pressure on this particular measure of convergence. This increase is itself additional evidence of divergence (diversification) rather than convergence. Therefore, proposition 6 is rejected.

## Analysis

We have empirically tested six propositions. Four have been confirmed, one has been rejected, and one could not be confirmed or rejected. In combination, the results of the four confirmed propositions reveal search strategies along the academic lifecycle. We know that, in the course of their academic career, scientists work in multiple finite research trails and that they often work in several problem areas at the same time. Thus, we provide scientometric and statistical proof for the conclusions of Zuckerman, Cole and Hagstrom. We also found that a scientist’s role in selecting problem areas changes over time and that entry into and exit from a problem area is associated with the potential for reputational gain. We found no evidence of convergence followed by divergence—quite the opposite—and could not prove or disprove the association between problem choice and career changes.

As PhDs, scientists work in one problem area, occasionally a second or third by the time they graduate. The first problem area is most likely suggested or provided by a supervisor. PhD research trails are often unconnected or relatively dissimilar to later research trails. In this phase, scientists work mainly as first author, which means that they do most of the work, even when they have not actually selected the problem area.

As postdoctoral researchers, scientists autonomously search for a personal niche that can develop into a coherent corpus. They expand their portfolio from one or two problem areas to as many as four or five and they extend their network of collaborators, publishing mostly in other or last author position. For postdoctoral researchers, diversification in problem areas and collaborators is a means to acquire a reputation. Expected and actual reputational gains explain entry into and rapid exit from problem areas. This suggests that the postdoctoral period is where scientists lay the foundations of their reputation and future career by constructing the core of their corpus, developing access to a community of peers, and building a social network of potential collaborators. The observed trends in average annual citations per publication suggest that most marginal gains are generated in the early years of work in a problem area. Parker et al. (2010) suggest the same thing: “highly cited research tends to be published in earlier career stages.” The postdoctoral period, where more than 40 % of a scientist’s research trails start, is where his reputation is cemented.

As professors, scientists work in a stable number of parallel trails. This may reflect that, in their responsibility as group leaders, publishing has become a group process in which they provide the grand design, while their PhDs carry out the work and their postdoctoral researchers search for new directions. Their work will be organised more according to project funding cycles and group size places a limit on their managerial span of control. The notion that the behaviour of scientists changes as they age is not new. Gingras et al. (2008) showed that as scientists age, they move towards last author positions and emphasise the collaborative aspects of research. Verbree et al. (2012a, b) find significant differences in the leadership and management activities of starting, experienced and nearly retiring research group leaders.

## Conclusions and discussion

In this paper we have developed a novel scientometric method to longitudinally map and statistically analyse the lifetime scientific output of individual scientists. Using this method, we have identified the search strategies of 43 condensed matter physicists along the academic lifecycle. In a nutshell, the results show that PhDs, postdoctoral researchers, and professors have their own specific search strategies. The search strategies of scientists along their academic lifecycle are all about strategic positioning. This involves selecting the right problem areas, developing a coherent portfolio or mix of problem areas, gaining access to communities of peers that can provide recognition, and developing from individual research to group leader. At no time is this more visible than during the postdoctoral phase when portfolios diversify and take shape, output increases, and researchers develop into leaders.

This study is not without limitations. We have examined a small sample from one scientific field, sufficient to develop scientometric and statistical methods with which to test a theoretical framework. Also, the selected scientists mostly (though not exclusively) have had longer careers and are accomplished, sometimes eminent, researchers. Our method needs more extensive testing. Specifically, our research agenda has three points. First, we plan to compare the search strategies of scientists in different fields and disciplines. This is where we scale up our method, comparing larger samples across different cognitive and institutional contexts and also testing its application to groups and institutions. Second, we need to focus on younger scientists, including some who dropped out of academia. A third question concerns the worldwide size and crowdedness of the problem areas that scientists select. Finally, the scientometric method must be combined with ethnographic and sociological methods to learn more about the behavioural dynamics of individual problem choice. Scientometric mapping can serve as an instrument during interviews to better understand the long-term strategies and short-term decisions of scientists in different stages of the academic lifecycle.

We believe that our method opens up new opportunities for research into the dynamics of agenda setting in science. It can provide new insights into the long-term dynamics and organisation of science and into the effects of policy and funding programmes. The results can provide university and S&T policy makers with a better understanding of the nature and extent of their leverage at the micro level. Also, the characteristics of search strategies can be expressed statistically as a set of evaluation indicators that takes the lifecycle of the subject into account.

Moreover, the results of this paper provide further support for the notion that science is a complex adaptive system. We have shown how individual rules and preferences play out in interaction with a community of peers and within the institutional organisation of science to produce complex individual portfolios. The development of institutional and national scientific portfolios can be seen as emergent from the problem choice of individual scientists who are constantly looking for new niches and communities where they can build their reputation, while progressing from PhD to research group leader along the academic lifecycle. By extension, communities and scientific specialties are also emergent. Community size is not constant, as has been suggested by Price and Wray (2010), but fluid. In time, the results of studies like this one should allow us to simulate the dynamics of science at different levels to better understand the effects and leverage of science policy.

## Footnotes

1 The Community Detection Tool is based on the original software produced by Blondel et al. It uses a fixed (i.e. non-random) starting point but does not as yet produce a measure for modularity. See Lancichinetti & Fortunato (2009) for a comparison of community detection algorithms.

2 A letter is not considered a citeable document in the calculation of journal impact factors. Letters are, however, part of the substantive contribution of a scholar to his field, they contain references to other literature, and they are cited. The note is no longer a document type in the Web of Science. Notes do, however, show up in longitudinal analysis. On average 1.0 % of all documents in our dataset is a letter and 3.3 % is a note.

3 We use the five citation databases of the Web of Science: Science Citation Index Expanded, Social Sciences Citation Index, Arts & Humanities Citation Index, Conference Proceedings Citation Index-Science, and Conference Proceedings Citation Index-Social Science & Humanities. Data were downloaded in March thru May 2011.
